# Oligonucleotide‐Based Modulation of Macrophage Polarization: Emerging Strategies in Immunotherapy

**DOI:** 10.1002/iid3.70200

**Published:** 2025-05-05

**Authors:** Hanfu Zhang, Yizhi Yu, Cheng Qian

**Affiliations:** ^1^ National Key Laboratory of Immunity & Inflammation, Institute of Immunology Naval Medical University Shanghai China; ^2^ School of Molecular Sciences University of Western Australia Crawley WA Australia

**Keywords:** immunotherapy, macrophage polarization, oligonucleotides, therapeutic strategies

## Abstract

**Background:**

Recent advances in immunotherapy have spotlighted macrophages as central mediators of disease treatment. Their polarization into pro‑inflammatory (M1) or anti‑inflammatory (M2) states critically influences outcomes in cancer, autoimmunity, and chronic inflammation. Oligonucleotides have emerged as highly specific, scalable, and cost‑effective agents for reprogramming macrophage phenotypes.

**Objective:**

To review oligonucleotide strategies—including ASOs, siRNAs, miRNA mimics/inhibitors, and aptamers—for directing macrophage polarization and their therapeutic implications.

**Review Scope:**

We examine key signaling pathways governing M1/M2 phenotypes, describe four classes of oligonucleotides and their mechanisms, and highlight representative preclinical and clinical applications.

**Key Insights:**

Agents such as AZD9150, MRX34, and AS1411 demonstrate macrophage reprogramming in cancer, inflammation, and infection models. Advances in ligand‑conjugated nanoparticles and chemical modifications improve delivery and stability, yet immunogenicity, off‑target effects, and formulation challenges remain significant barriers.

**Future Perspectives:**

Optimizing delivery platforms, enhancing molecular stability, and rigorous safety profiling are critical. Integration with emerging modalities—such as engineered CAR‑macrophages—will enable precise, disease‑specific interventions, and advance oligonucleotide‑guided macrophage modulation toward clinical translation.

## Graphical abstract

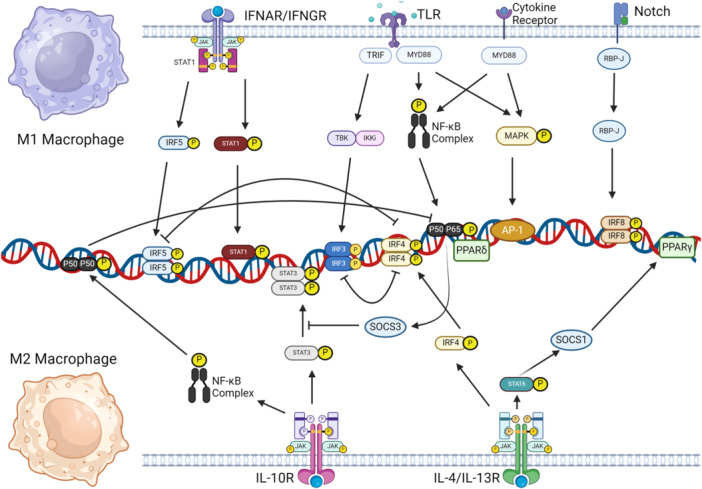

**Abbreviations**
ASOs: antisense oligonucleotides
CAR‐M: chimeric antigen receptor macrophage
CAR‐T: chimeric antigen receptor T cell
IFN: interferon
IL: interleukin
IRF: interferon regulatory factor
LPS: lipopolysaccharides
ManLAM: Mannose‐capped lipoarabinomannan
miRNAs: microRNAs
MyD88: myeloid differentiation primary response 88
RISC: RNA‐induced silencing complex
SELEX: Systematic Evolution of Ligands by EXponential Enrichment
siRNAs: small interfering RNAs
SIRPα: signal regulatory protein alpha
SOCS1: suppressor of cytokine signaling 1
Th1: T helper 1
TLRs: Toll‐Like receptors
TNF: tumor necrosis factor
TRIF: TIR domain‐containing adapter‐inducing interferon‐β

## 1 Background

### 1.1 Macrophage Polarization

Macrophages, integral components of the immune system, play important roles in tissue homeostasis [1, 2], immunity [3, 4], and inflammation regulation [5, 6]. These versatile cells can be polarized [7, 8, 9, 10] into distinct functional states. Such polarization results from a diverse array of stimuli [11, 12], leading to the differentiation of macrophages into two primary phenotypes: M1 (pro‐inflammatory) and M2 (anti‐inflammatory). Each phenotype further diversifies into subtypes, showcasing specialized functions and distinct signaling pathways. Notably, M1 macrophages exhibit pro‐inflammatory [13, 14] characteristics, promoting pathogen defense [7, 15] and antigen presentation [16, 17]. Conversely, M2 macrophages encompass a spectrum of subtypes (M2a, M2b, M2c, M2d) [18], playing crucial roles in wound healing [19], immune regulation [20], and anti‐inflammatory responses [21]. The intricate interactions among signaling pathways, cytokines, and transcription factors are pivotal in directing macrophage polarization, setting the stage for the development of precise immunotherapies and strategies for disease management. Thus, taking a closer look at these foundational nuance mechanisms and the functional nuances of various oligonucleotides is imperative.

#### 1.1.1 M1‐Type Macrophage Polarization and Regulated Signaling Pathways

**Figure 1:**
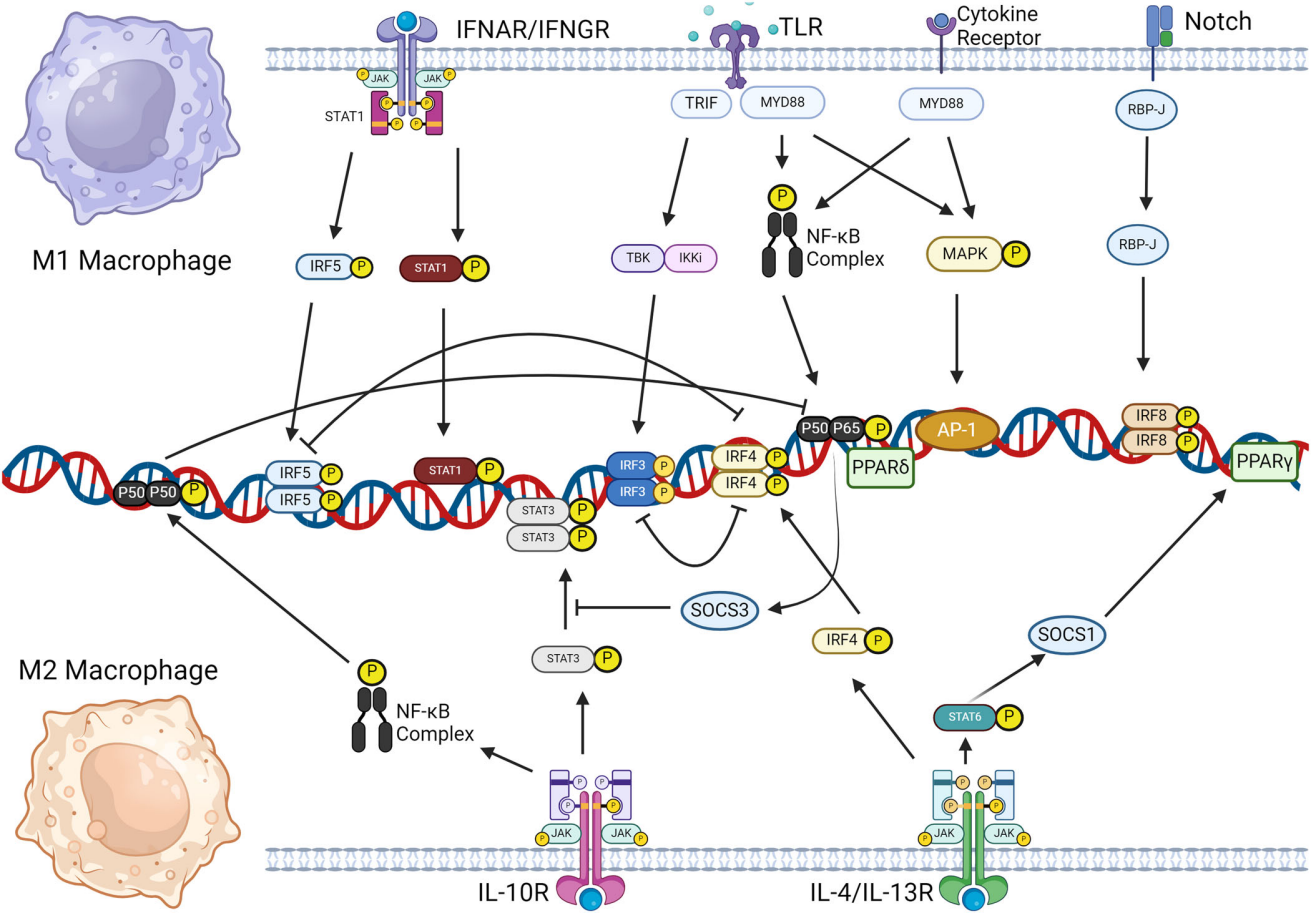
Receptors and signaling pathways that are involved in M1/M2 polarization. This schematic illustrates how different surface receptors (including TLRs, IFNAR/IFNGR, IL‐4/IL‐13R, IL‐10R, and the Notch receptor) and their downstream signaling cascades coordinate the polarization of macrophages into M1 or M2 phenotypes. M1 (classically activated) macrophages are typically driven by pro‐inflammatory signals. TLR ligation activates MyD88‐ and TRIF‐dependent pathways, culminating in the NF‐κB (p50–p65) complex and MAPK activation. Likewise, IFNAR/IFNGR engagement induces the phosphorylation of STAT1 and IRF3/IRF5, promoting transcription of genes responsible for inflammation (e.g., high IL‐12, TNF‐α). Notch signaling through RBP‐J can also reinforce M1 features. M2 (alternatively activated) macrophages emerge under anti‐inflammatory or tissue‐repair conditions. IL‐4/IL‐13 binding to their receptors triggers the JAK–STAT6 pathway, driving IRF4 and PPARγ activation and upregulation of M2‐specific genes (e.g., Arg1, Ym1). IL‐10R signals similarly recruit JAK–STAT3, reinforcing anti‐inflammatory effects. Notably, the NF‐κB p50–p50 homodimer also enhances M2 polarization by competing with, and thus inhibiting, the p50–p65 (pro‐inflammatory) complex. Transcriptional crosstalk is crucial: IRF4 can inhibit IRF3/IRF5‐mediated inflammatory pathways, while SOCS proteins (e.g., SOCS1, SOCS3) provide negative feedback that modulates JAK–STAT and NF‐κB. Arrows indicate activation, and blunt‐ended lines represent inhibition.

M1 macrophage polarization is governed by signals from interferon [22, 23], Toll‐like receptors (TLRs) [24], and interleukin receptors [22, 25], which activate the Janus Kinase‐Signal Transducer and Activator of Transcription, TIR domain‐containing adapter‐inducing interferon‐β (TRIF), and myeloid differentiation primary response 88 (MyD88) pathways (Figure 1). IFN‐γ from T helper 1 (Th1) cells drives M1 polarization [26, 27], with its receptor engaging Jak1 and STAT1 to induce inflammatory genes and major histocompatibility complex class II expression [28, 29, 30, 31], thus releasing interleukin (IL)‐12 [32], IL‐23, tumor necrosis factor (TNF)‐α [33], and other pro‐inflammatory interleukins triggered by interferon regulatory factor 5 (IRF5) [34]. TLR4 on macrophages responds to microbial stimuli like lipopolysaccharide (LPS), triggering TRIF [35, 36, 37] and MyD88 pathways [37, 38] leading to IRF3 activation for Type I interferon production and NF‐κB activation for pro‐inflammatory gene transcription [39, 40, 41, 42, 43]. The Notch‐RBP‐J pathway, amplified by IFN‐γ, further promotes M1 polarization via TLR4 [30, 44], while microRNAs like miR‐125a‐5p have been shown to regulate M1 polarization by silencing IRF4 pathway [45]. Collectively, M1 macrophages, shaped by a network of receptors and pathways, function in phagocytosis and antigen presentation, although their polarization can be modulated by signals like TLR4/IL‐10 interactions [46].

#### 1.1.2 M2‐Type Macrophage Polarization and Regulated Signaling Pathways

M2 macrophage polarization gives rise to four subtypes—M2a, M2b, M2c, and M2d—each distinguished by unique stimuli and surface markers but commonly expressing IL‐4 and IL‐10 [17, 47], which help define their immunoregulatory roles (Figure 1). M2a cells, emerging from IL‐4/IL‐13 interaction with IL‐4 receptor α, are central to Th2 immune responses, activating Jak1/Jak3 and STAT6, which regulate Arg‐1 transcription and macrophage fusion, and are influenced by IRF4 and suppressor of cytokine signaling 1 (SOCS1) [33, 48, 49, 50, 51, 52], contributing to M2 polarization, hence playing vital role in Th2 response. M2b macrophages, regulatory in nature, share markers with M1 but are notable for their high IL‐10 and unique markers like CD86 and SPHK1, and are regulated via LPS/IgG interactions and signaling pathways like TRIF, MyD88, and NF‐κB [53, 54, 55, 56, 57, 58, 59, 60]. Functionally, M2b macrophages play a crucial role in cardiovascular diseases [61], and modulation of M2b polarization may provide a novel therapeutic approach. M2c macrophages, or inactivated macrophages, respond to glucocorticoids and IL‐10, with a surface CD163 abundance, and act through Jak1‐STAT3, PI3K, and mitogen‐activated protein kinase pathways, suppressing M1 inflammation and aiding in tissue remodeling [22, 57, 62, 63, 64, 65]. Meanwhile, M2d macrophages, often tumor‐associated, produce factors promoting angiogenesis and tumor growth, are stimulated by IL‐6/TLR ligands, and their activation involves the IL‐6R/gp130 complex and Jak1/STAT3 pathway, leading to pro‐tumoral cytokine production [66, 67], thereby contributing to angiogenesis and metastasis [66, 67].

To summarize, while the four M2 macrophage classes vary in function, signaling patterns, and surface markers, they collectively contribute to macroscopic phenomena, including anti‐inflammation, matrix production, and wound repair.

### 1.2 Synthetic Oligonucleotides: Pioneering Therapeutics at the Genetic Frontier

Synthetic oligonucleotides, which range from 2 to 100 nucleotides [68], are chemically synthesized and play a pivotal role in genetic engineering, drug development, and diagnostics due to their specific targeting capabilities via Watson‐Crick base pairing [69]. These oligonucleotides encompass various types such as antisense oligonucleotides (ASOs), small interfering RNAs (siRNAs), miRNA mimics (agomirs), inhibitors (antagomirs), and aptamers, each with distinct mechanisms and applications, as discussed in subsequent sections.

**Figure 2:**
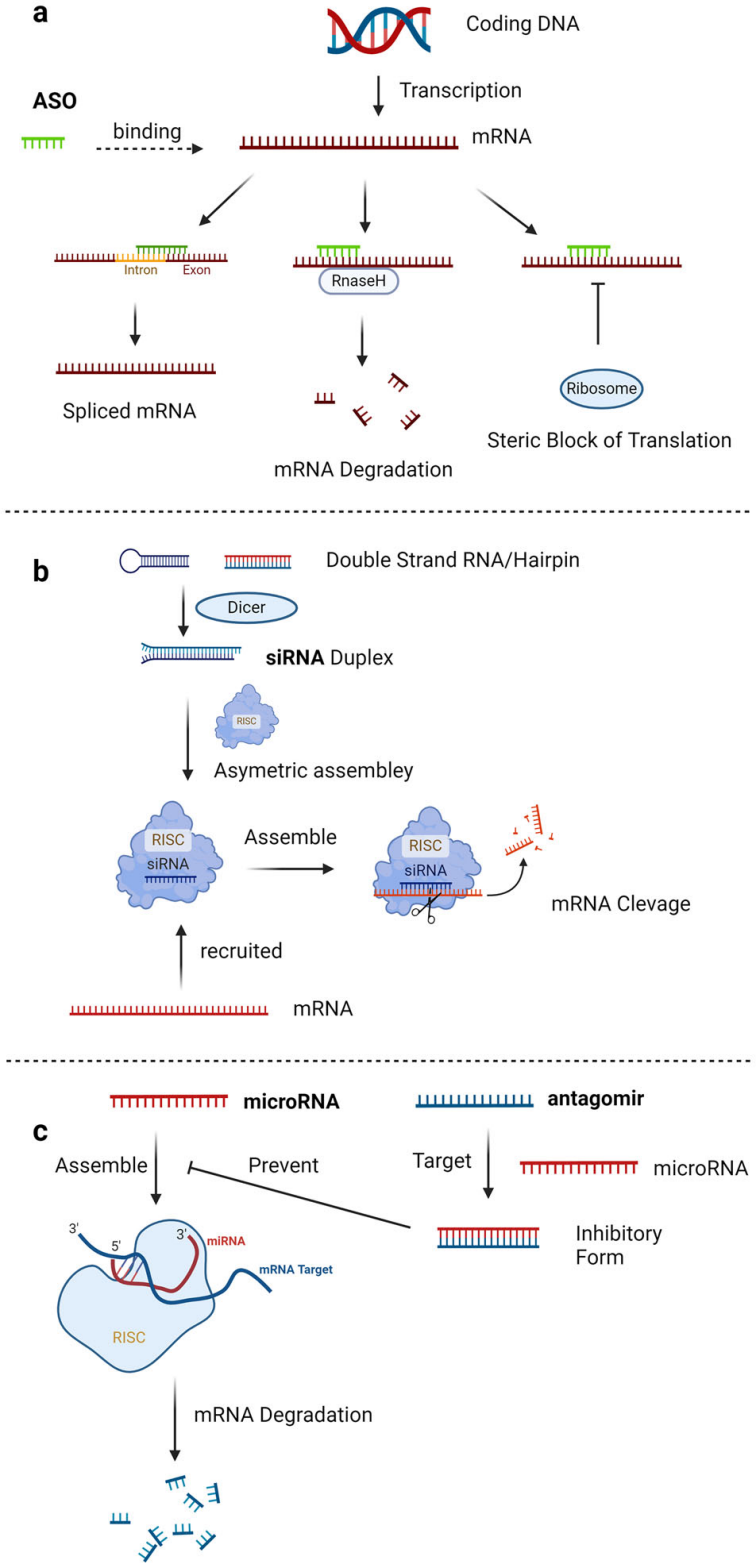
Functioning mechanisms of ASO, siRNA, and microRNA/antagomirs. Oligonucleotides working mechanisms. (a) Mechanism of ASO: ASOs work by binding to specific mRNA transcripts within a cell. This binding can result in the blockage of protein translation or the recruitment of RNase H, an enzyme that degrades the RNA strand of the RNA‐DNA duplex, effectively reducing the levels of the target protein. (b) SiRNA: Small interfering RNAs (siRNAs) function by pairing with complementary mRNA molecules in the cytoplasm. Once bound, they are incorporated into the RNA‐induced silencing complex (RISC), guiding the complex to the target mRNA. The RISC then cleaves the mRNA, preventing it from being translated into protein, thereby silencing gene expression. (c) MicroRNAs (miRNAs) and antagomirs: miRNA and antagomirs regulate gene expression post‐transcriptionally. miRNAs, loaded into RISC, bind imperfectly to target mRNAs, typically resulting in translational repression or mRNA degradation. Antagomirs, designed to be complementary to specific miRNAs, bind to and inhibit those miRNAs, preventing them from interacting with their mRNA targets and thus modulating the gene suppression effect of the miRNAs.

The mechanisms of different types of oligonucleotides vary as well. ASOs are typically 18–30 bases long [70], and bind to RNA to impede protein translation, functioning either by recruiting RNase H to degrade the target RNA [71, 72, 73] or by blocking splicing (Figure 2a) [74]. Meanwhile, siRNAs, which are double‐stranded molecules consisting of 21–25 nucleotides, function by directing the RNA‐induced silencing complex to degrade target mRNAs with complementary sequences, effectively silencing gene expression (Figure 2b) [75, 76, 77, 78]. MicroRNAs are usually 19–23 nucleotide long [79], and regulate gene expression by binding to mRNA's 3’ UTRs [80], affecting translation and stability [81, 82], with artifically synthesized agomirs enhancing and antagomirs inhibiting miRNA functions, thereby regulating gene expression post‐transcriptionally (Figure 2c). Both agomirs and antagomirs can be administered directly into human body [83]; however, they encounter the problem of degradation by ubiquitous nucleases [84]. This susceptibility to degradation poses a challenge for their therapeutic efficacy.

**Figure 3:**
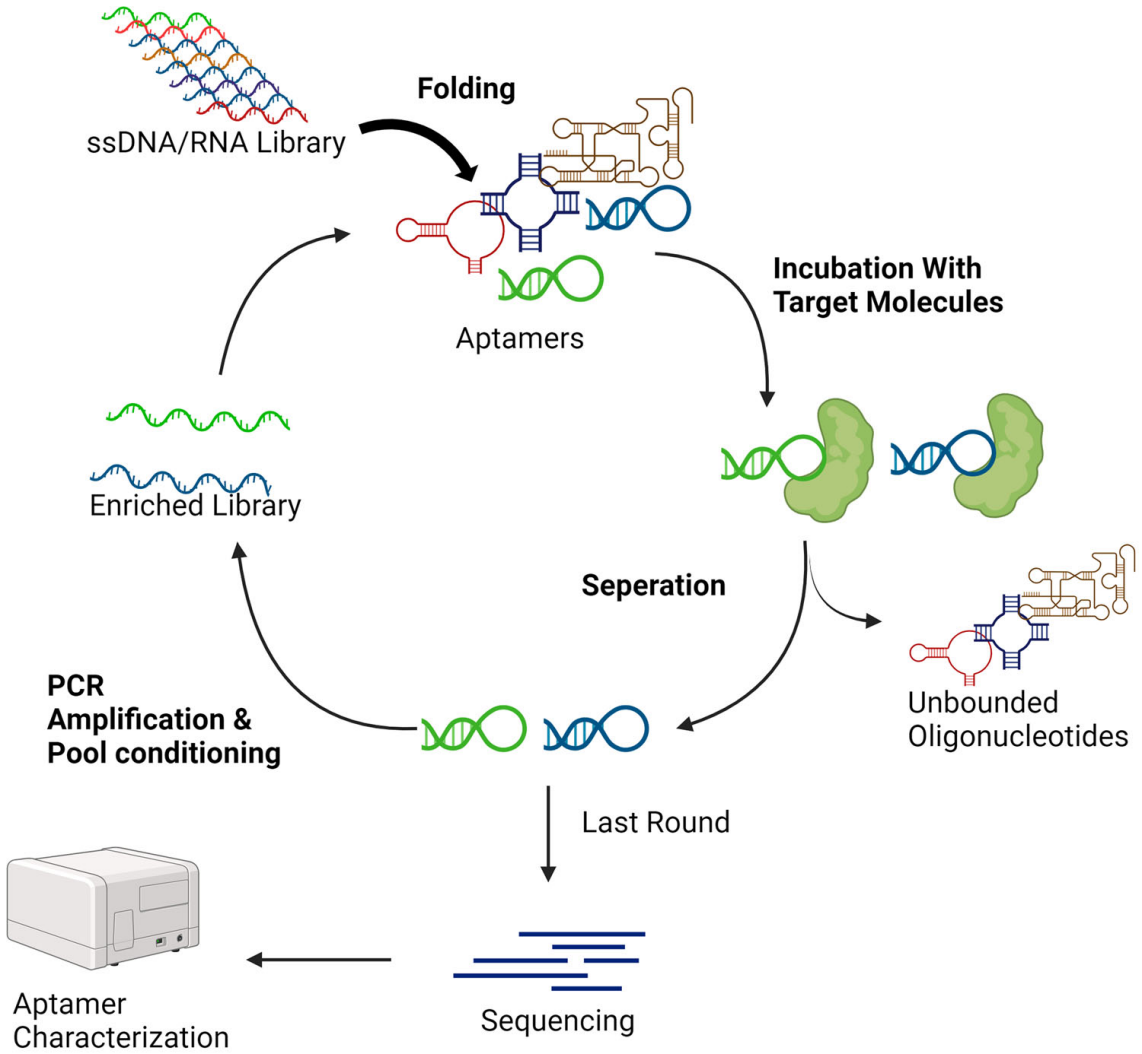
SELEX and aptamer generation. Starting with a diverse pool of RNA or DNA oligonucleotides, the sequence is incubated with the target molecule, allowing those with binding affinity to attach. Unbound sequences are then removed, and the bound oligonucleotides are eluted. These are subsequently amplified via PCR to generate a pool of enriched sequences. This selection cycle is repeated multiple times to increase the affinity and specificity of the aptamers. At the end of the process, high‐affinity aptamers are identified and sequenced, yielding candidates for further characterization and potential therapeutic or diagnostic applications.

Unlike ASO, siRNA or microRNA‐related oligonucleotides, spanning 12–80 base pairs, aptamers are oligonucleotides that achieve high specificity in binding to their targets through unique three‐dimensional structures, mirroring the precision of antibodies [85, 86, 87, 88]. They are selected through ‐processes of Systematic Evolution of Ligands by EXponential enrichment (SELEX) method, a multi‐step in vitro screening process that begins with the enrichment of oligonucleotides that bind specifically to target molecules (Figure 3), thus enabling precise targeting in diagnostics and therapy.

From the general description, it is obvious that each type of oligonucleotide serves a distinct function in regulating gene expression, thereby offering a comprehensive toolkit for both therapeutic and diagnostic applications across a multitude of disciplines. This showcases the extensive utility of synthetic oligonucleotides in modern biotechnology. As we delve deeper into specific examples, we will explore how these molecular tools are applied in real‐world scenarios, illuminating their potential to revolutionize treatments for diseases and enhance diagnostic precision. This exploration will not only highlight their effectiveness in current applications but also set the stage for their future development and integration into emerging biotechnological advancements.

### 1.3 Therapeutic Perspectives in the Application of Oligonucleotides to Modulate Macrophage Polarization

Macrophage polarization is crucial for both disease development and therapeutic strategies. Targeting macrophage states offers promising avenues for treating a wide array of diseases. For instance, enhancing M2 polarization could mitigate inflammatory and autoimmune diseases like rheumatoid arthritis (RA) [89] and lupus [90], whereas promoting M1 polarization may be effective against cancers [91] and infections [8]. Oligonucleotides, including microRNAs (miRNAs), present a targeted approach to modulate macrophage polarization due to their specificity, potential for reduced costs, and significant efficacy compared to conventional therapies [92], as nucleotide sequences can regulate macrophage behavior by targeting specific genes involved in the polarization process. The therapeutic potential of oligonucleotides in influencing macrophage states underlines the need for precise, disease‐specific interventions to harness the full benefits while minimizing risks. Future therapies might leverage oligonucleotides to reprogram macrophages toward desired states, offering new treatment modalities for various pathological conditions. This approach exemplifies the intricate interplay between immune modulation and therapeutic innovation, emphasizing the role of macrophage polarization in advancing medical treatments. Therefore, we will present cases in the next several sections, to discuss the future of using oligonucleotides to regulate macrophage polarization for therapies (Table 1).

**Table 1:** Overview of major oligonucleotide types and their mechanisms of action.

Oligonucleotide	Length (nt)	Typical mechanism	Key targets in macrophage polarization
Antisense oligonucleotides (ASOs)	18–30	1. RNase H‐mediated target RNA degradation 2. Steric hindrance/blocking of splicing/translation	miR‐155, STAT6, STAT3, MALAT1, NEAT1, etc
Small interfering RNAs (siRNAs)	21–25	1. RISC‐mediated cleavage of target mRNA 2. Translational repression	CD47, SIRPα, STAT3, IRF5, TNF‐α, etc
microRNA mimics and inhibitors (agomirs/antagomirs)	19–23	1. Mimic endogenous miRNA function (agomirs) 2. Competitively inhibit miRNA function (antagomirs)	miR‐155, miR‐21, miR‐34, miR‐125, etc
Aptamers	12–80	1. High‐affinity binding to proteins or small molecules via unique 3D structures 2. Intracellular or extracellular targeting	Nucleolin (AS1411), ManLAM, PD‐L1, various surface markers

## 2 Application of ASOs for Macrophage Regulation

In macrophages, ASOs modulate multiple signaling nodes or regulatory nucleic acids, influencing M1 or M2 macrophage differentiation and functions, thereby regulating processes like inflammation, immune response, and tissue repair. Current ASO targeting primarily involves RNA molecules including mRNA, tRNA, and regulatory miRNA. These have been exemplified in regulating macrophage polarization. Not only do these examples demonstrate effective regulation, but they've also been applied across various disease models and animal experiments, validating the potential of these ASOs as future therapeutics (Table 2).

**Table 2:** ASO that regulates macrophage polarization.

ASO	Target	Direction of polarization	Therapeutic effect	Reference
ASO miR‐155	miR155	M2+	M1 transformed to M2; obesity, diabetic cardiomyopathy, and acute liver failure models produce recovery from disease in mice	[93, 94, 95]
Oligo‐NEAT1	NEAT1‐MALAT1	M2+	Atherosclerosis and vascular inflammation are suppressed due to increased M2	[96]
ASO‐HuR	HuR	M2+	Increased expression of IL‐10, an anti‐inflammatory molecule, effectively relieves neuropathic pain	[97]
3GA‐494	miR‐494‐3p	M2+	Inhibits atheromatous plaque formation and stabilizes atheromatous plaque	[98]
Anti‐STAT6	STAT6	M1+	M1 ratio increased and showed excellent immune induction and cure in a symbiotic model of colorectal and hepatocellular carcinoma; significant improvement in tumor infiltration due to M2 TAM in NSCLC models	[99, 100]
Anti‐STAT3	STAT3	M1+	Improves immune activation and produces good efficacy in GL261 glioma after conjugating with CpG	[101]
ASO‐miR‐142‐5p	miR‐142‐5p	M1+	Significant therapeutic effects on mice in an asthma model with down‐regulated M2	[102]
3p‐125b‐ASO	miR‐125b	M1+	RIG‐I activated, M1 proliferation observed with good efficacy of immune activation in combined‐delivery	[103]
ASO miR‐21	miR‐21	M1+	Significantly elevated M1 quantity after co‐delivery with erlotinib and atezolizumab with enhanced therapeutic effect in MC38 cancer model	[104]
Anti‐circITGB6	circlTGB6	M1+	Reversal of circITGB6‐induced resistance to cisplatin‐based drugs in advanced ovarian cancer in mice due to the downregulation of M2	[105]

### 2.1 ASOs in M2 Polarization

In promoting M2 polarization, miR‐155 is a pivotal target under investigation. Elevated in various immune‐related diseases, ASOs targeting miR‐155 effectively modulate macrophage polarization, offering therapeutic potential in conditions such as tumors [106], inflammatory bowel diseases [107], autoimmune disorders [108], and infections [93]. Studies indicate miR‐155 overexpression shifts anti‐inflammatory M2 macrophages toward pro‐inflammatory M1, a reversible process, forming the basis for subsequent research [14, 94]. Utilizing different ASOs targeting miR‐155 expression effectively modulated various macrophage‐related disease models, demonstrating efficacy in treating obesity [109], diabetic cardiomyopathy [93], acute liver failure [95, 110], among others, validated through animal experiments, paving the way for future miR‐155 ASO applications.

Apart from miR155, other protein signaling targets also prove promising in M2 polarization regulation. For instance, targeting tRNA‐like sequences within the NEAT1‐MALAT1 gene cluster induces M2 macrophage polarization via GU‐rich ASOs, applied in atherosclerosis and vascular inflammation models [96], showing significant therapeutic effects. Targeting HuR RNA‐binding protein using ASOs elevates anti‐inflammatory molecule expression, enhancing M2 polarization [97], effectively alleviating neuropathic pain via nasal administration. Targeting miR‐494‐3p activates the Wnt signaling pathway, inhibiting M1 activation and inducing M2 transition, potentially impacting atherosclerotic plaque formation and stability [98]. These innovative approaches modulate immune responses, transitioning from pro‐inflammatory (M1) to anti‐inflammatory and tissue‐repairing (M2) macrophage phenotypes, highlighting a dual therapeutic action crucial for diseases marked by inflammation. Besides, intranasal delivery system for ASOs underscores a significant leap in drug administration, offering a noninvasive, direct pathway to the central nervous system, thereby overcoming the blood‐brain barrier and reducing systemic side effects. Also, the specificity of ASOs in targeting molecular pathways paves the way for precision medicine, presenting a targeted, effective, and safer treatment modality. Therefore, the strategy holds broad therapeutic potential, extending beyond neuropathic pain to include cardiovascular diseases, inflammatory conditions, and potentially cancer immunotherapy, by leveraging the nuanced roles of macrophage polarization in disease progression and resolution, signifying a transformative step in the development of molecular therapies.

### 2.2 ASOs in Immune Response Activation

On the other hand, by targeting immunosuppression‐related nucleotides, ASOs also can be the regulator toward the activation of immune responses, thus achieving the goal of immunotherapy. For example, ASOs targeting STAT6 [99, 100] and STAT3 [101] mRNA have been extensively studied in tumor immunotherapy, influencing M1/M2 characteristics, offering effective measures in cancer treatment strategies that allow macrophages to be proliferated toward the M1 polarization. Meanwhile, for immunosuppressive microRNAs such as miR142‐5p [102], miR‐125b [103], and miR‐21 [104], ASOs have been demonstrated to effectively enhance M1 expression. Therapeutically, ASOs targeting miR142‐5p exhibit therapeutic effects in asthma models, particularly in downregulating M2 markers, thereby achieving the goal of therapy; ASOs targeting miR‐125b activate the RIG‐I pathway, promoting M1 polarization, effectively combating breast cancer and metastasis; ASOs targeting miR‐21, when co‐delivered with specific drugs, significantly elevate M1 levels, showing potent inhibitory effects in tumor models. These examples of the application of ASOs targeting immunosupressive mRNA or microRNA underscores the potential for precise disease modulation across various models. By directly influencing macrophage polarization, ASOs offer a nuanced therapeutic strategy that not only aims to alter the underlying immune mechanisms of diseases but also enhances the efficacy of conventional treatments when used in combination.

In addition, ASOs targeting circular RNA, particularly circITGB6 [105], effectively halt M2 polarization, reversing platinum‐based drug resistance in late‐stage ovarian cancer models, presenting a novel approach for cancer therapy. However, limited evidence exists linking ASOs targeting circular RNA to macrophage polarization control, necessitating further research to ascertain their significance.

Currently, AstraZeneca's AZD9150, targeting STAT3 mRNA, is undergoing clinical trials, displaying safety, pharmacokinetics, and preliminary antitumor activity in pancreatic cancer. Numerous other ASOs targeting CXCL12, NOX‐A12 promoting M1 polarization, and ISIS‐304801 targeting ApoCIII regulating inflammation in the pancreas are also in various stages of clinical trials, demonstrating their potential in modulating macrophage polarization for specific diseases. Despite little data has been revealed currently, it still worth to look forward to their safety and efficacy.

In conclusion, ASOs present a promising avenue for modulating macrophage polarization on both regulating M1 and M2 polarization, yet the full clinical viability of this regulatory mechanism remains uncertain. Demonstrating effectiveness in targeting diverse RNA molecules and proteins to influence M1/M2 macrophage characteristics, ASOs offer potential therapeutic interventions across various disease models. While preclinical studies showcase encouraging results, their translation into robust clinical applications encounters significant challenges. Ongoing clinical trials, notably AZD9150 targeting STAT3 mRNA, hint at safety and preliminary efficacy in pancreatic cancer treatment but lack definitive evidence for widespread clinical adoption. The transition from promising preclinical outcomes to tangible clinical benefits necessitates thorough validation, considering complexities like in vivo dynamics, off‐target effects, delivery systems, and long‐term safety profiles. Despite positive trends, ASOs’ role in transformative therapeutic approaches for macrophage modulation awaits rigorous validation through further research, extensive clinical trials, and comprehensive safety assessments to bridge the gap between potential and clinical utility.

## 3 The Role of siRNA in the Regulation of Macrophage Polarization and Prospects for the Treatment of Related Diseases

From the previous discussion of the impact of the pathways involved in macrophage polarization, it is not difficult to find that the use of RNA interference technology can achieve a significant downregulation of the expression of the target protein and thus achieve the purpose of regulating macrophage polarization through the regulation of the expression of a certain signal in the pathway involved in polarization and affecting the interaction of the other nodes of the pathway, which will then regulate macrophage polarization. Therefore, theoretically, we believe that RNAi technology can provide new ideas for the treatment of many macrophage‐polarization‐related diseases.

### 3.1 siRNAs in M1 Polarization

It was found that CD47 plays an important role in macrophage polarization as the “don't eat me signal.” By binding to signal regulatory protein alpha (SIRPα) on the macrophage surface, CD47 confers the ability to fight against phagocytosis by macrophages. siRNA blockade of CD47 has been shown to polarize macrophages into inflammatory M1 cells, which in turn exert an antitumour effect [111]. This finding makes siRNAs targeting CD47 or SIRPα important products for the regulation of polarization. In recent years, with the discovery of more pathways, the mechanisms by which macrophage polarization is regulated have become more complex, and a large number of siRNAs have been shown to be able to convert macrophages in a pro‐inflammatory or anti‐inflammatory direction. The following table contains some representative cases of siRNA regulation of macrophage polarization in recent years (Table 3):

**Table 3:** siRNA that regulates macrophage polarization.

siRNA	Pathway regulated	Target of siRNA	Direction of polarization	Reference
Anti‐lncRNA260	PI3K/Akt, JAK‐STAT	IL28RA	M2+	[112]
FOXO1‐siRNA	PI3K/Akt	FOXO1	M1−	[113]
CHOP/AMPKα1‐siRNA	PI3K/Akt	C/EBPβ, AMPKa1	M1+, M1−	[114]
SNAIL1‐siRNA	PI3K/Akt, TGF‐β	SNAIL1	M1+, M2−	[115]
Notch1R‐siRNA	Notch	Notch‐1	M1−, M2+	[116, 117]
Si‐SIRPα; si‐SHP1	Notch	SIRPa, SHP1	M1−, M2+	[118]
STAT3/HIF‐1α si‐RNA	JAK‐STAT	STAT3, HIF‐1a	M1+, M2−	[119]
SART1‐siRNA	JAK‐STAT	STAT6/PPAR	M2−	[120]
IRF5‐siRNA	TLR4	IRF5	M1−, M2+	[121, 122]
p65‐siRNA	NF‐kB	P65	M1−, M2+	[123]
siIRF4	IRF4	IRF4	M1+, M2−	[124]
Mecp2‐siRNA	IRF4	Mecp2	M1+, M2−	[125]
TNF‐a‐siRNA	NF‐kB	TNF‐a	M1−, M2+	[126]
siAKT1, siSOCS1	NF‐kB	AKT1, SOCS1	M1+, M2−	[127]

In terms of promoting polarization in the M1 direction, several experiments have demonstrated that siRNAs can activate macrophages by silencing specific inhibitory target signaling proteins, thereby inducing a stronger pro‐inflammatory response. For example, silencing of zinc finger protein (SNAIL1) significantly increased the levels of marker mRNA in M1‐polarized macrophages in undifferentiated colonies, which in turn increased the levels of pro‐inflammatory factors and resulted in a significant reduction in the number of TGF‐β‐induced anti‐inflammatory macrophages, M2, which were insensitive to exogenous TGF‐β stimulation [115]. In addition, delivery of siRNAs targeting STAT3 and HIF‐1α was shown to be effective in increasing M1 numbers in in vitro experiments and achieved excellent antitumour therapeutic effects in in vivo experiments [119]. Meanwhile, C/EPBβ [114], which plays an important role in the PI3K/AKT pathway, PPARγ [120], which has a role in the induction of M2 in the JAK/STAT pathway, IRF4 [124] and MECP2 [125], which have an inducible role in the IRF pathway, and Akt1/SOCS1 [127], which inhibits M1 activation in NF‐κB, were all shown to be effective in increasing the number of M1 macrophages in experiments. Given the successful examples involving various mechanisms and targets, it is clear that siRNAs hold significant promise as agents in future immunotherapies, particularly for promoting M1 polarization and enhancing inflammatory responses, which can play crucial roles in antitumor and anti‐infection efforts. Their potential for clinical translation warrants further exploration. However, a notable gap in these studies is the lack of systematic cellular‐level monitoring, leading to scarce reports on potential side effects and dosage requirements. In addition, related animal studies often lack sufficient scale or replication to draw robust conclusions. Thus, while siRNAs exhibit great promise, there remains a multitude of challenges in siRNA‐related translational research that must be meticulously addressed through comprehensive experiments before progressing toward clinical application.

### 3.2 siRNAs in M2 Polarization

A large number of experiments exist on promoting polarization in the M2 direction as well, demonstrating that siRNAs have important anti‐inflammatory roles and are able to promote the polarization of M2‐associated macrophages. Among them, lncRNAs, which have been extensively studied in recent years, have received focused attention as regulatory targets of siRNAs. In terms of regulatory effects on M2 polarization, it was found that by binding to and downregulating the expression of lncRNA260, siRNAs could further activate the PI3K/Akt pathway by reducing the alternative splice variants of IL28RAV2, which would in turn increase the induction level of M2 and exert important anti‐inflammatory effects [112]. Conversely, siRNAs targeting mRNAs that interfere with the FOXO1 protein, crucial for M1 polarization, failed to elevate key M1 signals (IL1β, IL6) in RAW264.7 macrophages, with or without protease‐activated receptor 2 stimulation, which highlights FOXO1's potential as a significant anti‐inflammatory target of siRNA‐therapy [113]. In addition, in the Notch signaling pathway, which is important for M1 polarization, siRNAs targeting important positive feedback signals in this pathway, such as Notch1 [116, 117], SIRPα [118], and SHP1 [118], have also been shown to inhibit M1 polarization and enhance M2 polarization. For targeting the IRF family, it has been shown that its siRNAs are also able to complete the inhibition of M2 polarization [121, 122], but the specific mechanism is not yet known; moreover, by targeting the classical M1 pathway activation nodes p65 [123] and TNF‐α [126], siRNAs are able to inhibit M1 activation very effectively and increase the percentage of M2 anti‐inflammatory cells in the total colony.

Given the complexity of macrophage polarization, which involves multiple pathways and mechanisms—such as SNAIL1's ability to regulate several pathways simultaneously or IRF/TNF‐α‘s involvement in various polarization directions—we cautiously refrain from prematurely attributing equivalent translational value to the siRNAs discussed, pending more definitive experimental evidence, particularly from specific disease animal models. For instance, si‐AMPKα1 has been applied in atherosclerosis research, while Notch1‐siRNA, si‐TNF‐α, and p65‐siRNA have been explored in RA models [117, 123, 126]. Similarly, siRNAs targeting STAT3/HIF‐1α have been utilized in an OS‐RC‐2 renal cancer cell model [119]. Furthermore, si‐Sart1 [120], si‐IRF5 [122], and si‐Mecp2 [125] have shown efficacy in models of idiopathic pulmonary fibrosis and severe acute pancreatitis, underscoring their potential as drugs for further investigation. These findings highlight the necessity of exploring siRNAs’ translational value through detailed, disease‐specific experimental validations.

## 4 Prospects for microRNA Mimetic (Agomir) and microRNA Inhibitor (Antagomir) to Regulate Macrophage Polarization and Thereby Treat Disease

In recent years, extensive research on miRNAs has revealed their intricate involvement in regulating macrophage polarization. Degradation poses a significant challenge for miRNA‐based therapies, as miRNAs and their derivatives, agomirs and antagomirs, are prone to rapid breakdown in biological systems [128, 129]. This issue, alongside the non‐specificity of miRNAs targeting multiple mRNA targets and affecting crucial signaling pathways, complicates their therapeutic use. Despite promising regulatory effect over macrophage polarization, practical application hurdles such as ensuring stability, specificity [130, 131], and precise delivery [132] remain. Addressing these challenges is crucial for advancing miRNA therapies into clinical practice. Moreover, both double‐stranded and single‐stranded RNA molecules serve as substrates necessary for TLR recognition and activation within macrophage endosomes [133, 134, 135]. Although the typical length of an agomir as a double‐stranded RNA might not meet the 46 bp requirement for Toll‐Like Receptor 3 (TLR3) dimer‐forming activation [136], reports suggest that TLR3 can be activated by siRNAs [133], similar to double‐stranded oligomeric RNAs, hinting at potential activation by double‐stranded agomirs. Likewise, as a single‐stranded RNA, antagomir might activate the recognition ability of TLR7/8 for single‐stranded RNA [137], possibly leading to adverse immune responses and interfering with polarization determination.

In this context, we present examples of recent essential applications of agomirs/antagomirs in modulating macrophage polarization, integrating these with established miRNA target clinical trial cases. However, our search results, despite the voluminous reports from numerous experiments, permit only selective listings, offering rather preliminary insights largely confined to miRNA‐related modulation in specific disease models over the last 3 years. Thus, we opt to succinctly report main clinical trial outcomes and macrophage polarization‐related information in the following paragraph.

Regarding clinical trials, numerous miRNA targets linked to macrophage polarization have been researched. Although the in vivo specificity, safety, and stability issues of miRNA have constrained most major clinical trials to early stages, necessitating further research and optimization. Despite this, several known cases of agomirs and antagomirs, showcasing evident stimulus effects, have entered clinical trials. For instance, MRX34, a miR‐34 mimic promoting M1 polarization, was clinically evaluated for various cancers [138, 139] (NCT02862145, NCT01829971) but was terminated due to severe immune side effects. Conversely, miR‐16 agomir MesomiR‐1 underwent a phase I clinical trial for malignant pleural mesothelioma and non‐small cell lung cancer evaluation [140] (NCT02369198), showing good tolerability in preliminary results, yet subsequent results and follow‐up studies remain unpublished. Antagomirs, relatively smoother in clinical applications, such as SPC3649 (Miravirsen), an miR‐122 antagomir assisting M2 activation for anti‐inflammatory effects, have been employed in several clinical trials evaluating therapies for hepatitis C [141]. However, despite conducting three phase II clinical trials (NCT01200420, NCT01872936, and NCT01727934) to validate its inhibitory effect on the hepatitis C virus, no experiments have published validated results to date. In addition, RG‐012 (Lademirsen, miR‐21 antagomir) completed two crucial early‐stage clinical trials (NCT03373786 and NCT02136862) without published outcomes [142, 143, 144]. Other miRNA antagomirs capable of modulating macrophage polarization, such as miR‐92a (MRG‐110), miR‐103/107 (RG‐125/AZD4076), miR‐122 (RG‐101), miR‐155 (MRG‐106/Cobomarsen), and AMT‐130 (miHTT), have entered initial clinical trials. However, similar to prior experimental projects, these early clinical trials offer good safety but limited result about efficacy has been published. Consequently, we currently lack substantial grounds to engage in a detailed discussion concerning the application of agomirs/antagomirs based on these clinical trials, let alone their actual effects on regulating macrophage polarization as demonstrated by experimental data.

To summarize, agomirs and antagomirs represent crucial branches for regulating macrophage polarization via oligonucleotides, backed by multiple signaling pathways and regulatory mechanisms, providing a relatively sturdy theoretical foundation for their continued development in this regard. Multiple agomirs and antagomirs have entered the pivotal stage of early clinical trials, potentially demonstrating substantial breakthroughs in the coming years, thus showcasing commercial viability for more drugs of this nature. However, the prospective applications of such miRNA‐related drugs must consider not only immunogenicity and length‐related innate immune responses, but also complexities such as nucleic acid degradation, which may lead to inadequate therapeutic efficacy or a higher incidence of severe adverse events in early clinical phases. Nevertheless, despite the current complexities, we maintain that after overcoming these hurdles, agomirs and antagomirs represent pivotal developmental directions for regulating macrophage polarization.

## 5 Aptamer: An Alternative Macrophage‐Regulating Oligonucleotide

As the specificity allows aptamer to recognize various in vivo targets, including proteins, small molecules [145], cell surface receptors [146], viruses [147], bacteria [148], and more, remarkably, in terms of protein recognition, some aptamers can rival antibodies in precision [149]. Aptamers serve a dual role by directly regulating protein activity within signaling pathways to influence macrophage polarization and by facilitating detection and synthesis of conjugates/nanoparticles, acting as crucial markers or guides. Given the variance in surface marker proteins across macrophage types, aptamers offer a swift method for tracking macrophage polarization states. For example, through SELEX, Sylvestre et al. [150] discovered the A2 Aptamer, which uniquely binds with high affinity to M2 macrophages, shows some affinity toward M0 and monocytes, but not to M1 macrophages, suggesting CD14 as A2's potential target. A2's efficient internalization by M2 macrophages highlights its promise in drug delivery, positioning it as an essential targeting molecule. This advancement suggests the potential of A2 in facilitating drugs that modulate M2 polarization, offering a novel approach to treating associated diseases. The development and application of more A2‐like aptamers are expected to refine the regulation of macrophage polarization, guiding macrophages toward M1 or M2 phenotypes, thereby enhancing therapeutic outcomes.

Beyond their indirect effects on macrophage polarization, aptamers are increasingly used directly as inducers or therapeutic agents to alter macrophage states. Illustrated by examples in the accompanying table (Table 4), various delivery methods and application strategies have demonstrated clear benefits in animal studies, providing valuable insights into aptamer utilization in this arena.

**Table 4:** Aptamers that regulate macrophage polarization.

Aptamer	Target	Form of delivery	Direction of polarization	Therapeutic effect	Reference
BM2	ManLAM	Direct	M1+	Triggered ManLAM–CD44 signaling, and enhanced M1 macrophage and Th1 activation	[151]
AS1411	Overexpressed nucleolin/M0 macrophage	Gold‐based polyvalent spherical aptamer	M1+	Significantly improved induction of Au‐PSA, increased M1 level at lower radiation doses and produced stronger killing of 4T1 tumors	[152]
SYL3C	EpCAM	Aptamer‐anchored spherical nanoparticle	M1+	Increased level of M1, significant growth inhibition and cytotoxicity in HT‐29 tumors, with downregulation of signals such as variant p53 and bcl‐2	[153]
ZXL1	ManLAM	Direct	M1+	Enhances IL‐1β and IL‐12 mRNA expression, decreased IL‐10 level, upregulated NOS level after induction of ManLAM, promotes the killing effect toward Mtb	[154]
EpCAM‐AsiC	EpCAM/tumor gene	Aptamer‐linked siRNA chimeras (EpCAM‐AsiCs)	M1+	Inhibited tumor progression and promoted tumor‐infiltrating immune cell functions	[155]
AS1411/PD‐L1 aptamer	Overexpressed nucleolin/PD‐L1	Chimeric aptamer‐engineered M1 Mφ	N/A	Expands the range of nongenetic macrophage cell engineering strategies	[156]
DNA aptamer A2 (CD14 targeting)	Proteins with single amino acid mutations	Direct	N/A	Specifically targeting M2 macrophages	[150]
MTX	TNF‐α	MTX‐loaded Tapt‐tFNA polyplexes	M2+	Promote the expression of M2 macrophages and inhibit inflammatory factor infiltration, hence achieving therapeutic effect to autoimmune disease such as RD	[157]
Synovial‐meniscal (S‐M) aptamers	Synovial/meniscal bispecific	Gelatin methacryloyl hydrogel (GelMA)	M2+	Recruits endogenous synovial and meniscal cells and promotes fibrocartilage regeneration for meniscus repair with better biocompatibility with lower inflammatory response	[158]
FKN‐aptamer	Fractalkine (FKN)	Aptamer‐functionalized hydrogels	M2+	Significant local increase in CD206+ M2‐like macrophages and recruitment of anti‐inflammatory subpopulations to the site of injury without the need for delivery of foreign proteins or cells	[159]

### 5.1 Aptamer in M1 Polarization

The mannose‐capped lipoarabinomannan (ManLAM) serves as a prime target for aptamers in regulating M1 polarization [151, 154]. This lipid protein, intricately linked to the CD44 signaling pathway, becomes a specific binding site for aptamers. By targeting ManLAM, aptamers can block its receptor interaction, directly promoting the polarization and activation of M1 macrophages and thus kickstarting immune responses. An illustrative example is the use of targeted aptamer therapy against BM2, which significantly boosted the protective efficacy of the Bacillus Calmette–Guérin vaccine vaccine against Mycobacterium bovis in both mouse and monkey models [151]. The enhanced protection was attributed mainly to the increased polarization of M1 and CD4+ T cells. Building on this, Pan et al.'s study [154] delved into the mechanisms through which the ZXL1 aptamer targets ManLAM. Their findings indicated that the ZXL1 aptamer not only mitigates the immune suppression mediated by CD44 but also augments the expression and production of IL‐1beta and IL‐12 mRNA and cytokines in ManLAM‐treated macrophages. In addition, it elevates inducible nitric oxide synthase and PPARγ expression while decreasing IL‐10 production. These multifaceted actions highlight the ZXL1 Aptamer's potential and value as a vaccine adjuvant, promising to amplify immune responses effectively.

Aptamers excel in precision targeting, serving as invaluable tools for indirectly influencing M1 polarization by facilitating the introduction of drugs that enhance M1 activity. In the realm of antitumor therapies, aptamer‐loaded nanoparticles designed to activate and destroy macrophages have shown promising results. For example, AS1411 aptamer [152], when integrated into multi‐affinity gold nanoparticles (Au‐PSA), triggers immune responses capable of eliminating 4T1 tumor cells by targeting the overexpressed nucleolin, a key tumor antigen. This strategic targeting allows for more efficient photo‐thermal tumor therapy, achieving heightened M1 levels with reduced radiation exposure, and thereby enhancing the tumor's eradication. Similarly, the SYL3C aptamer [153], by targeting the tumor antigen EpCAM, influences macrophage polarization, leading to reduced levels of mutant p53 and bcl2 and curbing tumor growth. Furthermore, employing the EpCAM aptamer alongside siRNAs that suppress multiple genes overexpressed in breast cancer—and coupling this with CD47 siRNA to boost M1 levels—achieves a stronger antitumor effect [155]. While these aptamers do not directly regulate polarization like CD47 or ManLAM aptamers, they demonstrate impressive potential in augmenting local M1 functionality for future therapeutic strategies. Nonetheless, the novel nature of this therapeutic approach introduces challenges in the regulatory approval and assessment process, potentially slowing the clinical application of aptamer‐based strategies in modulating macrophage polarization in the immediate future.

The exploration of chimeric antigen receptor T cell (CAR‐T) therapy has unveiled significant potential alongside challenges. To navigate these obstacles, novel CAR immunotherapies, such as CAR‐natural killer and the innovative CAR‐macrophage (CAR‐M) therapies, have been developed. A recent breakthrough involves engineering M1 macrophages (ApEn‐M1) [156] by chemically coupling aptamers AS1411/programmed death‐ligand 1 to create CAR‐like structures similar to chimeric antigen receptors. These engineered M1 cells showcase potent tumor‐targeting capabilities and exhibit remarkable cytotoxicity in models of breast cancer xenografts and lung metastases, alongside activating a range of immune cells in mice, thus underscoring their potential in antigen presentation and immune activation. Given the precision of aptamers in targeting and the versatile functionalities imparted by macrophage polarization, it is envisaged that this approach could extend beyond mere cytotoxic actions, unlike traditional CAR‐T therapies [156]. By manipulating initial in vitro polarization directions, we believe that this strategy could generate not only CAR‐M1 cytotoxic macrophages aimed at various antigens or surface markers but also develop CAR‐M2 anti‐inflammatory macrophages. These innovations hold promising application prospects in tissue and organ repair, treatment of autoimmune diseases, and prevention of transplant rejection, highlighting a transformative step forward in immunotherapy that leverages the unique advantages of macrophage plasticity.

### 5.2 Aptamer in M2 Polarization

Moreover, Aptamers also hold vast potential in regulating M2 polarization. They target key molecules involved in immune activation, blocking M1 generation, enhancing M2 proportions, thereby modulating anti‐inflammatory responses. Ongoing experiments and recent reports indicate promising applications. For instance, TNF‐α targeted aptamer in tetrahedral framework nanoparticles with methotrexate, a RA drug, successfully enhances M2 polarization and macroscopically improves various issues, including systemic toxicity [158]. Synovial and meniscal targeted aptamers encapsulated in hydrogels recruit endogenous synovial and meniscal cells to defect sites, increasing M2 polarization, promoting biocompatibility, reducing inflammation, thereby facilitating meniscus in‐situ regeneration and cartilage protection [157]. In addition, encapsulating FKN (CX3CL)‐aptamer in hydrogels enriches endogenous FKN in a mouse excisional skin wound model, increasing M2 macrophages for tissue repair [159]. These application cases highlight the value of aptamers in activating M2. They not only leverage the targeting ability of aptamers but also demonstrate exceptional results and promising prospects by effectively applying aptamers’ blocking capabilities to protein‐targeted therapeutic approaches. Importantly, an additional consideration arises regarding the size of the oligonucleotides used. Larger oligonucleotides (> 40 nucleotides), such as aptamers or double‐stranded RNAs (dsRNAs), can be perceived as foreign entities by the innate immune system, thereby acting as potent “induction stimuli” rather than mere “modulators.” Specifically, dsRNAs longer than 45 nucleotides can trigger TLR3‐mediated signaling cascades, leading to robust type I interferon responses and a pronounced M1 macrophage (Th1/M1) polarization profile [160]. This phenomenon underscores that the initial host defense response, mediated via pathways like TLR3, may precede and overshadow any subtle regulatory effects intended by the oligonucleotide, thereby influencing the final polarization outcome. Engineered macrophages have highlighted the cholesterol–AS1411 (26‐mer DNA aptamer) ‐anchored M1 macrophages (CAM1) as a promising strategy for cellular immunotherapy. By anchoring cholesterol–AS1411 aptamers on the surface of M1 macrophages, CAM1 can be generated without genetic modification or cellular damage. To address this potential interpretive bias, future analyses could directly compare the polarization directions induced by oligonucleotides of different lengths.

While this discussion cannot encapsulate the full extent of aptamers’ capabilities, ongoing and future research is expected to unveil a wide spectrum of therapeutic strategies and mechanisms, both anticipated and unforeseen. Among these, AS1411 is notably the most advanced, having progressed to phase II clinical trials, although comprehensive results are yet to be published (www.clinicaltrials.gov). This underscores the evolving landscape of aptamer research and its promising trajectory in clinical applications.

## 6 Conclusion and Future Directions

Oligonucleotide‐based strategies to modulate macrophage polarization offer a promising avenue for disease treatment, from inflammatory disorders to cancer. The capacity of ASOs, siRNAs, miRNA agomirs/antagomirs, and aptamers to precisely influence macrophage states underscores their potential as versatile therapeutic tools. Recent advances in chemical modifications, nanocarrier design, and delivery systems have begun to mitigate issues such as rapid degradation and off‐target effects, bringing these interventions closer to clinical viability. Ensuring stable and targeted oligonucleotide delivery to macrophages in vivo requires more sophisticated vector systems and precise ligand‐engineering to navigate complex biological environments. It is also critical to elucidate how oligonucleotide length, structural motifs, and chemical modifications influence innate immune activation. Addressing these challenges through comprehensive preclinical evaluations, safety profiling, and long‐term follow‐up studies will guide the rational development of oligonucleotide‐based therapeutics and foster their integration into future clinical standards.

While the promise of oligonucleotide‐based macrophage modulation is evident, several limitations currently hinder clinical translation. Achieving stable, targeted delivery of oligonucleotides to macrophages in vivo remains a primary challenge, as conventional delivery systems often suffer from premature degradation, suboptimal biodistribution, and unintended accumulation in nontarget tissues. In addition, off‐target effects, immunogenicity concerns, and the need for repeated dosing raise safety and tolerability issues. To address these limitations, future research must prioritize these aspects: development of biocompatible nanocarriers, ligand‐conjugated aptamers, and stimuli‐responsive vehicles to enhance macrophage‐specific uptake. Systematic evaluation of how oligonucleotide length, chemical modifications, and structural conformations influence innate immune activation and unwanted gene silencing. Comprehensive preclinical and clinical evaluations to establish durable therapeutic benefits, minimize adverse events, and ensure scalability in clinical manufacturing.

By confronting these limitations through concerted research efforts, we can refine oligonucleotide‐based therapies, ultimately transforming them into clinically viable and impactful treatments for a variety of macrophage‐associated diseases.

## Author Contributions

**Hanfu Zhang:** investigation, writing – original draft. **Yizhi Yu:** funding acquisition, project administration, writing – review and editing. **Cheng Qian:** investigation, writing – review and editing.

## Conflicts of Interest

The authors declare no conflicts of interest.

## Data Availability

All data generated or analyzed during this study are included in this article. All schematics were created using BioRender (https://www.biorender.com/), and the authors authorize their publication.
